# Initial surgical management of injuries to the upper extremities for patients with multiple and/or severe injuries – a systematic review and clinical practice guideline update

**DOI:** 10.1007/s00068-026-03256-8

**Published:** 2026-07-08

**Authors:** Till Berk, Nadja Könsgen, Philipp Lechler, Sebastian Imach, Michael Schär, Dan Bieler, Klemens Horst, Frank Hildebrand, Käthe Goossen

**Affiliations:** 1 Department of Orthopedics, Trauma and Reconstructive Surgery, University Hospital RWTH Aachen, Aachen, Germany; 2 Institute for Research in Operative Medicine (IFOM), Witten/Herdecke University, Cologne, Germany; 3 Center for Orthopaedics and Trauma Surgery, University Hospital Giessen and Marburg, Location Marburg, Baldingerstraße, 35043 Marburg Germany; 4 Department of Trauma and Orthopedic Surgery, Cologne-Merheim Medical Center (CMMC), University of Witten/Herdecke, Cologne, Germany; 5 Department of Orthopaedic Surgery and Traumatology, University of Bern, Inselspital, Bern, Switzerland; 6 Department of Trauma Surgery and Orthopedics, Reconstructive Surgery, Hand Surgery, Burn Medicine, German Armed Forces Central Hospital Koblenz, Koblenz, Germany; 7 Department of Trauma, Hand- and Reconstructive Surgery, University Hospital Münster, Albert-Schweitzer-Campus 1, 48149 Münster, Germany

**Keywords:** Amputation, Guideline, MESS score, Nerve injuries, Polytrauma, S3, Severely injured, Surgery, Timing, Upper extremities, Vascular injuries

## Abstract

**Purpose:**

Our objective was to update the evidence-based and consensus-based recommendations for the initial surgical management of upper extremity injuries in patients with suspected multiple and/or severe injuries based on current evidence. This guideline topic is part of the 2025 update of the German Guideline on the Treatment of Patients with Multiple and/or Severe Injuries.

**Methods:**

MEDLINE and Embase were systematically searched to September 2024. Further literature reports were obtained from clinical experts. Randomised controlled trials (RCTs) or observational studies reporting risk-adjusted outcomes were included if they compared early versus delayed surgical treatment for fractures, vascular injuries, or nerve injuries affecting the upper extremities in patients with multiple and/or severe injuries. Studies comparing limb salvage versus amputation or comparing amputation criteria for the upper extremities were also included. We considered patient-relevant outcomes such as mortality and limb salvage, as well as the sensitivity and specificity of scores for predicting upper extremity amputation. Risk of bias was assessed at the outcome level using ROBINS-I for observational studies and AMSTAR-2 for systematic reviews. We used available meta-analyses if possible; alternatively, we synthesised the evidence narratively. We used GRADE to rate the certainty of evidence. Expert consensus was used to develop recommendations and determine their strength.

**Results:**

Among 2498 records screened for eligibility, four studies were included. Observational studies addressed the timing of humeral fixation, the timing of revascularisation, or compared limb salvage with amputation. A systematic review evaluated scoring systems for mangled upper limb salvage. Four recommendations were modified, and one consensus-based recommendation was added. All five recommendations achieved strong consensus.

**Conclusion:**

The timing of surgical intervention for upper extremity injuries in polytrauma patients should be individualized, based on the patient’s physiological status, injury pattern, and the risks and benefits of early versus delayed surgery. Although early surgery may improve functional outcomes and reduce complications, adequate patient stabilization is essential before proceeding. Limb salvage should be attempted for patients with a MESS score of less than seven, provided the patient is sufficiently stable and depending on the condition of the amputated limb.Although the guideline addresses patients with multiple and/or severe injuries, the scarce evidence, heterogeneity of the included studies and the limited representation of true polytrauma cohorts may restrict the applicability of the findings to the broader critically injured trauma population.

## Introduction

Upper extremity injuries occur frequently in polytrauma cases [1–3]. These injuries, while often less life-threatening than those in other body regions, can significantly impact patient outcomes, including prolonged hospitalization and increased disability [4]. The complexity of upper extremity injuries in polytrauma patients necessitates a multidisciplinary approach to their management. Factors such as the type of fracture (open vs. closed), presence of blood vessels or nerve damage, and the patient’s overall physiological status can influence treatment decisions and outcomes [5]. Early surgical intervention, coupled with comprehensive rehabilitation, is crucial for optimizing functional recovery and minimizing long-term complications [5]. Even though upper extremity injuries in polytrauma patients may not significantly affect overall survival, they play a critical role in the patient’s functional recovery and quality of life. Timing can be a pivotal factor influencing patient outcomes. Furthermore, early stabilization of extremity fractures in polytrauma patients has been linked to reduced durations of intensive care unit stays and mechanical ventilation, suggesting a more efficient recovery process [2]. In cases of distal radius fractures, early surgical intervention (within two weeks) has been associated with improved functional outcomes, as measured by the Disabilities of the Arm, Shoulder, and Hand (DASH) scores, compared to delayed surgery [3].

Consequently, the available contemporary evidence on the management of upper extremity injuries in patients with severe trauma was systematically reviewed. This body of evidence prompted a comprehensive revision of the corresponding chapter in the updated German S3 guideline for the treatment of severely injured patients. The updated recommendations aim to reflect current best practices, optimize interdisciplinary trauma care, and improve functional outcomes while ensuring consistency with modern trauma management principles.

## Methods

This guideline topic is part of the 2025 update of the German Guideline on the Treatment of Patients with Multiple and/or Severe Injuries [6]. The guideline update is reported according to the RIGHT tool [7], the systematic review part according to the Preferred Reporting Items for Systematic Reviews and Meta-Analyses (PRISMA) 2020 reporting guideline [8]. The development and updating of recommendations followed the standard methodology set out in the guideline development handbook issued by the German Association of the Scientific Medical Societies (AWMF) [9]. The update included a substantial revision of the previous guideline methodology.

### PICO questions and eligibility criteria

Population, intervention, comparison (PIC) questions were retained from the previous guideline version. In addition, the participating professional societies involved in guideline development were asked to submit new PIC questions. The final set of PIC questions was selected in the guideline steering group. The key guideline questions for this topic area were:

In adult patients (≥ 14 years) with polytrauma and/or severe injuries including upper extremity injuries, does early surgical intervention improve patient-relevant outcomes compared with delayed surgery? Does limb salvage, amputation, or replantation improve patient-relevant outcomes, and which decision criteria for limb salvage should be applied?

The full set of predefined PIC. The study eligibility criteria in the PICOS format are shown in Table 1.

**Table 1:** Predefined eligibility criteria

Population	Adult patients (≥ 14 years) with polytrauma and/or severe injuries and injuries to the upper extremities; for a full definition, see Figure S1 (Online Resource).
Intervention /comparison	• Intervention: early surgical treatment of fractures, vascular injuries, or nerve damage; amputation; replantation of a severed limb; defined decision criteria for amputation • Comparison: delayed surgical treatment of fractures, vascular injuries, or nerve damage; limb salvage; no replantation of a severed limb; different decision criteria for amputation (or no defined criteria)
Outcomes	Pre-defined list of outcomes: • Mortality • Quality of life (SF-12, SF-36, or similar) • Functional outcome (global scores, in particular the Disability of Shoulder, Arms, and Hand Questionnaire (DASH) and the Constant-Murley Score (CS)) • Severe/life-threatening complications (including infections, e.g., osteomyelitis) • Change in body temperature • Improvement in physiological condition • Time to haemostasis, blood loss (transfusion requirement, transfusion volume) • Length of hospital stay • Length of ICU stay/ICU-free days/time to ICU • Unplanned return to operating room • Neurological outcome/cognitive function • Disability • Morbidity • Emergency surgery • Limb salvage • Compartment syndrome • Pain (VAS, CRPS critical regional pain syndrome if applicable)
Study type	Study design (stepwise inclusion): 1. systematic reviews 2. RCTs 3. observational studies with risk-adjusted outcomes
Language	English or German
Other inclusion criteria	• full text of study published and accessible • study matches predefined PICO question
Exclusion criteria	• study already included in previous guideline version*

* The start date for searches was set to the date of the last search of the previous guideline version. The full-text eligibility of previously included studies was reassessed based on revised selection criteria. Whenever the new searches identified studies already included in the previous guideline version, these duplicate studies were excluded

### Literature search

An information specialist systematically searched for literature in MEDLINE (Ovid) and Embase (Elsevier) on 12 September 2024. The search strategies contained index (MeSH/Emtree) and free text terms for the population and intervention. Because this is a guideline update, the date of the previous search (15 May 2009) was used as the start date. Table S2 (Online Resource 1) provides details for all searches. Clinical experts were asked to submit additional relevant references.

### Study selection

Study selection was performed independently in a three-step process using the predefined eligibility criteria (1). Incorporation of single reviewer screening using the ‘exclusion criteria with no loss in sensitivity’ reported by Nama et al. [10]. References retrieved from database searches were filtered using Endnote search filters for the following exclusion criteria: children, case reports, animals, non-systematic reviews (see Table S3, Online Resource). The filtered references were screened by a single reviewer (2). Title/abstract screening by two reviewers (KG, NK) independently in Rayyan [11] of all references that could not be excluded with certainty in step (1) and those that were not pre-filtered, and (3) independent full-text screening by two reviewers of all articles deemed potentially relevant at the title/abstract level in Endnote (Endnote, version: 20 [Software], Clarivate, Boston, Massachusetts, USA. https://endnote.com/). Full texts cited in earlier guideline versions were checked for eligibility based on the updated inclusion criteria. Disagreements were resolved through consensus or by consulting a third reviewer (TB). The reasons for full-text exclusion were recorded (Table S4, Online Resource).

### Assessment of risk of bias and certainty of evidence

We critically assessed the methodological quality of the included studies at outcome level using the *Assessing the Methodological Quality of Systematic Reviews* (AMSTAR-2) tool [12], the Cochrane Risk of Bias 2.0 tool [13] for RCTs, the ROBINS-I tool [14] for comparative registry studies and cohort studies, and the *Quality In Prognosis Studies* (QUIPS) tool for prognostic studies [15]. The assessment was carried out independently by two reviewers (KG, NK) during a pilot phase, then performed by one person and verified by a second. Any discrepancies were discussed until consensus was reached.

The certainty of the evidence was assessed at outcome level using the *Grading of Recommendations Assessment*,* Development and Evaluation* (GRADE) methodology [16]. GRADE assessment was performed by one person (KG) and verified by a second (NK). Any discrepancies were discussed until consensus was reached. For RCTs or when using the ROBINS-I or QUIPS tool to assess the risk of bias, the recommended initial rating is ‘high’ [17, 18]. The certainty was rated down when there were concerns related to risk of bias, inconsistency, indirectness, imprecision, publication bias, or other factors affecting certainty.

### Data items and data extraction

A predefined data set was collected for each study, consisting of study characteristics (study type, setting), patient selection criteria and characteristics of relevant subgroups (age, gender, injury severity score (ISS), or other relevant scores), intervention and comparator, relevant outcomes, statistical model and variables used for risk adjustment, and effect measures for relevant outcomes. Data were extracted into a standardised data extraction sheet by one reviewer (KG) and checked by another (NK).

### Selection and prioritisation of outcomes

The selection and prioritisation of outcomes followed the GRADE process [19, 20]. A list of 73 potentially relevant outcomes was obtained from core outcome sets for trauma [21–25] and the evidence tables of the previous versions of the German polytrauma guideline. The coordination team for the prehospital section of the German polytrauma guideline (*n* = 5 clinical experts) pre-selected 13 outcomes from this list for relevance in the prehospital management of major trauma in an online survey hosted on LimeSurvey Community Edition (Version 5.x) [26]. The lead clinical expert (TB) was asked to add topic-specific outcomes and surrogate outcomes as required, resulting in a list of 17 outcomes (Table 1, Outcomes). Their importance was rated by the clinical experts involved in updating this guideline topic (TB, KH, PL, and SI) on a scale of 1 (less important) to 9 (critical for decision-making) in an online survey using LimeSurvey. The mean importance rating for each outcome was used for the development of recommendations (see Table S5, Online Resource).

### Synthesis of studies

The evidence was synthesised at the outcome level. The evidence profiles for each PIC question were created directly in the MAGICApp (https://app.magicapp.org/#/guideline/8812) in the ‘Evidence’ section. In case results from at least 2–3 studies with a low or moderate risk of bias were available for an outcome, further studies with a high or critical risk of bias were not included in the evidence synthesis for this outcome.

For most outcomes, the evidence was summarised narratively. We used existing systematic reviews where possible. If at least three clinically comparable studies with a suitable effect measure (e.g. adjusted odds ratios) were available, we planned to perform a meta-analysis if possible. Meta-analyses of dichotomous data were planned in SPSS Statistics (version 29) using a random effects model with restricted maximum likelihood (REML) method and Knapp-Hartung adjustment as an estimator for the variance between the studies. Heterogeneity was planned to be assessed using precision intervals.

### Development and updating of recommendations

For each PICO question, the following updating options were available: (1) the recommendation of the preceding version remains valid and requires no changes (“confirmed”); (2) the recommendation requires modification (“modified”); (3) the recommendation is no longer valid or required and is deleted; (4) a new recommendation needs to be developed (“new”). An interdisciplinary expert group of clinicians and nurses with expertise in the management of severe trauma and acute care (‘guideline group’) created Evidence to Decision (EtD) tables according to the GRADE method [27]. The steering group decided to limit the EtD tables to the domains ‘benefit and harm’, ‘certainty of evidence’, ‘values and preferences’, and ‘feasibility’. Based on the EtD tables, the clinical experts (TB, PL, SI, MS, KH, FH, and DB) and methodologists (KG, NK) drafted recommendations and proposed grades of recommendation (Table 2). The patient representatives reviewed recommendations and EtD tables.

**Table 2:** Grading of recommendations

Symbol	Grade of recommendation	Description	Wording (examples)
⇑⇑	A	strong recommendation	“use …”, “do not use …”
⇑	B	recommendation	“should use …”, “should not use …”
⇔	0	open recommendation	“consider using …”, “… can be considered”

In the absence of eligible evidence, recommendations were made based on clinical experience and expert consensus in cases where the guideline group felt a statement was required due to the importance of the topic. These were not graded and instead labelled as good (clinical) practice points (GPP). For GPPs, the strength of a recommendation is presented in the wording shown in Table 2.

### Consensus process

The Guideline Group finalised the recommendations during web-based, structured consensus conferences on 15 January 2025 via Zoom (Zoom, Version: 5.x [Software], Zoom Video Communications, Inc., San José, California, USA. https://zoom.us). A neutral moderator facilitated the consensus conferences. Voting members of the guideline group were delegates of all participating organisations, including clinicians, emergency medical services personnel, nurses, and patient representatives (delegates of the patient organisation *Unfall-Opfer-Bayern e.V.*). Each organisation had one vote. Guideline methodologists attended in a supporting role. Members with a moderate, thematically relevant conflict of interest abstained from voting on recommendations, members with a high, relevant conflict of interest were not permitted to vote or participate in the discussion. A member of the expert group for the guideline topic presented recommendations. Following discussion, the guideline group refined the wording of the recommendations and modified the grade of recommendation as needed. Agreement with both the wording and the grade of recommendation was assessed by anonymous online voting using the survey function of Zoom. Abstentions were subtracted from the denominator of the agreement rate. Consensus strength was classified as shown in Table 3.

**Table 3:** Classification of consensus strength

Description	Agreement rate
strong consensus	> 95% of participants
consensus	> 75 to 95% of participants
majority approval	> 50 to 75% of participants
no approval	< 50% of participants

Recommendations were accepted if they reached consensus or strong consensus. For consensus recommendations with ≤ 95% agreement, diverging views by members of the Guideline Group were detailed in the background texts. Recommendations with majority approval were returned to the expert group for revision and further discussion at a subsequent consensus conference. Recommendations without approval were considered rejected.

### Peer review

During a four-week consultation phase, the recommendations and background texts were submitted to all participating organisations for review. Comments were collected using a structured review form. The results were then assessed, discussed and incorporated into the text by the guideline coordinator with the relevant expert group for each guideline topic.

The guideline was adopted by the executive board of the German Trauma Society on 7 November 2025.

### Quality assurance

The guideline recommendations were reviewed for consistency between guideline topic areas by the steering group. Where necessary, changes were made in collaboration with the clinical leads for all topic areas concerned. The final guideline document was checked for errors by the guideline chair and methodologist.

## Results

**Fig. 1:**
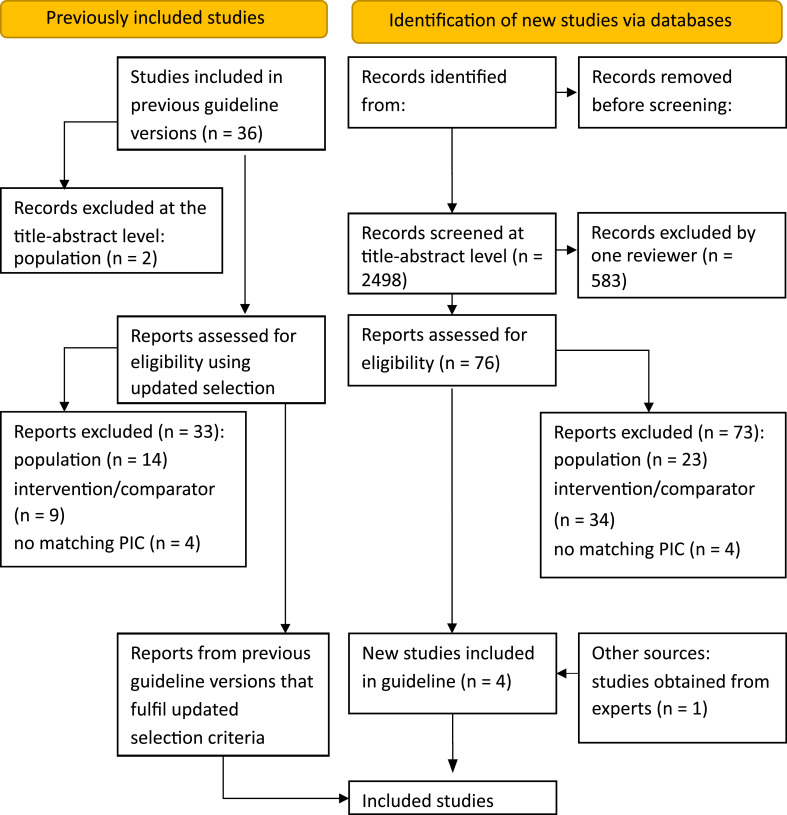
Modified PRISMA 2020 flow diagram showing the systematic literature search and selection of studies

The database searches identified 2498 unique records (Fig. 1). In addition, 36 references from the previous guideline version were screened. Additional records were obtained from clinical experts. In total, 4 studies were eligible for this update [28–31]. Of these, 3 references were identified in the new search [28–30] and 1 reference was obtained from experts [31]. A list of excluded full-text articles can be found in Table S4 (Online Resource).

### Characteristics of included studies

Study characteristics and main outcomes are presented in Table 4. Full details are provided in Table S6 (Online Resource). We included three observational studies conducted in the USA [28–30] and one systematic review [31]. They were mostly from national or regional registers. In one study, the population were military personnel with combat injuries [29].

**Table 4:** Characteristics of included studies

Study, reference	Design, setting	Population (*N*)	Intervention / comparison
Chipman 2023 [28]	National registry USA, 2007–2016	7908 patients with upper extremity arterial injury 59% penetrating trauma	Reperfusion time ≤ 90 min vs. >90 min
Mitchell 2019 [29]	Secondary analysis of a retrospective cohort study USA, 2003–2007	137 patients with unilateral major upper-extremity injuries	Amputation vs. limb salvage
Ritter 2023 [30]	National registry USA, 2010–2015	3059 patients with humeral shaft fractures (55% with ISS ≥ 14)	Humeral fixation on day 1 vs. day 2 or later
Yoneda 2024 [31]	Systematic review search covered 1985 to 2022	10 studies with 338 patients in the meta-analysis for the *Mangled Extremity Severity Score* (MESS)	MESS ≥ 7 vs. <7

### Risk-of-bias and GRADE certainty assessment

The risk of bias was assessed using ROBINS-I for 6 outcomes. Overall, it was moderate for 2, serious for 3, and critical for 1 outcome. The main reasons for serious/critical risk of bias were confounding (4 outcomes) and selection of participants (1 outcome). The included systematic review was rated as moderate using the AMSTAR 2-tool. The summary of findings and GRADE certainty assessment at outcome level is presented in Table 5.

**Table 5:** Summary of findings and GRADE certainty assessment

Comparison: Early vs. delayed fixation of humerus shaft fractures in polytrauma patients				
**Outcomes**	**No. of participants (studies)**	**Relative effects ** **adj. HR (95% CI)**	**Certainty of the evidence (GRADE)**	**Comments**
ICU days (4: important)	2664 patients (1 study [30])	day 1: 0.88 (0.69 to 1.12) vs. day ≥ 2: 0.89 (0.70 to 1.15) ^*a*^	⨁⨁⨁◯ moderate due to imprecision	The timing of humeral fracture fixation (on the day of the accident or later) probably makes little or no difference to the length of ICU stay.
Length of hospital stay (4: important)	2664 patients (1 study [30])	day 1: 0.96 (0.85 to 1.08) vs. day ≥ 2: 0.96 (0.85 to 1.08) ^*b*^	⨁⨁⨁◯ moderate due to imprecision	The timing of humeral fracture fixation (on the day of the accident or later) probably makes little or no difference to the length of hospital stay.
**Comparison**: Prognostic ability of severity scoring systems for salvage or amputation of mangled upper limbs				
**Outcomes**	**No. of participants (studies)**	**Prognosis of amputation**,** MESS cutoff ≥ 7 (95% CI)**	**Certainty of the evidence (GRADE)**	**Comments**
Limb salvage (8: critical)	338 patients (10 studies [31])	sensitivity: 0.95 (0.69–0.99) ^*c*^ specificity: 0.81 (0.65–0.91) ^*d*^	⨁⨁⨁◯ moderate due to inconsistency	A MESS score ≥ 7 probably does not accurately predict the need for amputation of a severely injured upper limb. A MESS < 7 probably predicts successful salvage of a severely injured upper limb.
**Comparison**: Amputation vs. limb salvage following major upper-extremity trauma				
**Outcomes**	**No. of participants (studies)**	**Relative effects**, **adjusted (95% CI)**	**Certainty of the evidence (GRADE)**	**Comments**
Functional outcome (SMFA total dysfunction) (8: critical)	137 patients (1 study [29])	adj. MD − 2.95 (–8.78 to 2.87) ^*e*^ all sub-scores n.s.	⨁◯◯◯ very low due to risk of bias, indirectness and very serious imprecision	We are uncertain whether amputation or limb salvage improved functional outcomes in patients with major upper-extremity trauma.
Pain interfering with activity (CPG) (5: important)	137 patients (1 study [29])	adj. OR 1.05 (0.35 to 3.16) ^*f*^	⨁◯◯◯ very low due to risk of bias, indirectness and very serious imprecision	We are uncertain whether amputation or limb salvage affected pain interfering with usual activities in patients with major upper-extremity trauma.
**Comparison**: Early vs. delayed revascularization in upper extremity arterial injury				
**Outcomes**	**No. of participants (studies)**	**Amputation**,** p-value (unadjusted time-to-event analysis)**	**Certainty of the evidence (GRADE)**	**Comments**
Amputation rate (8: critical)	5407 patients (1 study [28])	significantly less unadjusted amputation in the ≤ 90 min group: *p* = 0.007 ^*g*^ (1.8% overall amputation rate in the study population)	⨁◯◯◯ very low due to very serious risk of bias and imprecision	We are uncertain whether early surgical revascularisation (< 90 min) reduced the amputation rate compared to delayed revascularisation (≥ 90 min).

CPG, Chronic Pain Grade (scale of 0-100, where 0 is best / 100 is worst disability). n.s., not significant. SMFA, Short Musculoskeletal Function Assessment (scale of 0-100, where 0 is best / 100 is worst function). ^*a*^ Reference group not clearly defined in the publication, possibly “no fixation”; Cox proportional hazards regression models adjusting for AIS, GCS, ISS, age, and gender; patients without ICU stay excluded from analysis ; ^*b*^ Reference group not clearly defined in the publication, possibly “no fixation”; Cox proportional hazards regression models adjusting for AIS, GCS, ISS, age, and gender; ^*c*^ Sensitivity: the probability that patients needing amputation had a MESS ≥ 7; ^*d*^ Specificity: the probability that patients with MESS ≥ 7 needed amputation (81% specificity suggests that limb salvage was achieved in at least 20% of the patients whose MESS was above the threshold); ^*e*^ multivariable linear regression adjusting for age, military rank, combat experience, social support, presence of lower extremity injury and months to interview; ^*f*^ multivariable logistic regression adjusting for age, military rank, combat experience, social support, presence of lower extremity injury and months to interview; ^*g*^ Time-to-event curves for amputation modelled using the Kaplan-Meier method followed by the log-rank test in order to assess for in-between group significant differences; not adjusted for confounders

### Recommendations

Four recommendations were modified, and one new recommendation were developed based on the updated evidence and expert consensus (Table 6). The Evidence-to-decision assessments are p.

**Table 6:** List of recommendations with grade of recommendation and strength of consensus

No.	GoR, GRADE ^a^	Evidence, consensus	Recommendation (translated from German)	Status 2025
3.8.1	0 ⇔ moderate ^*b*^ ⨁⨁⨁◯	[30] 100%	Consider deciding on the timing of surgical repair of long bone fractures in the upper extremities on an individual patient basis.	Modified
3.8.2	B ⇑ moderate ⨁⨁⨁◯	[31] 100%	In patients with a critically injured upper extremity, limb preservation should be pursued when the MESS score is < 7. For patients with a MESS score ≥ 7, the decision between limb salvage and amputation should incorporate patient-specific factors.	Modified
3.8.3	B ⇑ very low ⨁◯◯◯	[28] 100%	In cases of arterial injury to the upper arm, revascularization should be performed as early as possible.	Modified
3.8.4	GPP	100%	Upper extremity nerve injuries with discontinuity should be managed promptly, taking the patient’s overall condition into account. Management should adhere to the principles outlined in the S3 guideline “Treatment of Peripheral Nerve Injuries” (AWMF registry number 005/010). ^*c*^	New
3.8.5	GPP	100%	When nerve injuries with discontinuity are directly associated with a fracture, treatment should be performed in conjunction with definitive fracture stabilization.	Modified

GoR, grade of recommendation. ^*a*^ GRADE certainty rating across all outcomes. ^*b*^ All available outcomes were important, but not critical. ^*c*^ https://register.awmf.org/de/leitlinien/detail/005-010

## Discussion

In this systematic review and prospective clinical practice guideline update, recommendations for the initial surgical management of upper extremity injuries in patients with multiple and/or severe trauma were developed. Based on the current literature with strict inclusion criteria and interdisciplinary consensus on the balance of benefit and harms, certainty of the evidence, patient values and preferences, and feasibility, the following five key recommendations were identified:

1. Consider deciding on the timing of surgical repair for upper extremity long bone fractures on an individual patient basis.
  The recommendation regarding the timing of surgery was based on a U.S. registry study including 2,664 patients, specifically in adult patients (> 14 years) with polytrauma or trauma-related severe injury and fractures of the long bones of the upper extremity [30]. No differences were observed between operative fixation of humeral fractures on day 1 (adjusted HR 0.88 [0.69–1.12]) versus day > 2 (0.89 [0.70–1.15]; reference in both cases: no surgery). In summary, the current evidence does not demonstrate relevant differences between early (day 1) versus delayed operative management of humeral fractures after trauma regarding short-term outcomes (duration of intensive care and hospital stay). However, evidence is lacking for critical benefit and harm endpoints, including quality of life, limb preservation, mortality, and severe or life-threatening complications [30].

In accordance with Advanced Trauma Life Support (ATLS) principles, life-threatening sources of haemorrhage in severely injured patients should be identified and controlled without delay, including bleeding associated with long tubular bones such as the tibia and femur [32]. In patients, who are hemodynamically unstable, injuries of the upper extremity are commonly managed with temporary immobilization using casts or splints during the initial resuscitation phase, with definitive surgical treatment deferred until physiological stabilization has been achieved [32].

Early surgical intervention, typically within 24 to 72 h post-injury, has been associated with several benefits. In the context of upper extremity reconstruction, early microsurgical procedures have demonstrated lower rates of surgical revisions and flap loss compared to delayed interventions. This may be attributed to the avoidance of trauma-induced thrombocythemia, which peaks in the second week post-injury and can increase the risk of thrombotic complications [1].

Furthermore, early stabilization of extremity fractures in polytrauma patients has been linked to reduced durations of intensive care unit stays and mechanical ventilation, suggesting a more efficient recovery process [2]. In cases of distal radius fractures, early surgical intervention (within two weeks) has been associated with improved functional outcomes, as measured by the Disabilities of the Arm, Shoulder, and Hand (DASH) scores, compared to delayed surgery [3]. Despite the benefits, early surgery is not universally advantageous. In patients who are hemodynamically unstable or have ongoing systemic issues, immediate surgery may exacerbate their condition, leading to increased morbidity and mortality. The concept of ‘Damage Control Orthopaedics’ (DCO) advocates for initial temporary stabilization, such as external fixation, followed by definitive surgery once the patient’s physiological status has improved [4]. Additionally, controlled delays in surgery have not been shown to increase adverse events. A study indicated that patients who underwent delayed surgery for upper extremity fractures did not experience higher rates of emergency department revisits or hospital readmissions. Moreover, these patients had shorter inpatient stays compared to those who underwent earlier surgery [5]. When interpreting this recommendation, the underlying evidence base must be carefully considered in order to adequately adapt the comprehensive clinical picture of polytrauma as a systemic condition to the individual patient context.

2. In patients with a critically injured upper extremity, limb preservation should be pursued when the MESS score is < 7. For patients with a MESS score ≥ 7, the decision between limb salvage and amputation should incorporate patient-specific factors.

Regarding amputation, the recommendation was based on a meta-analysis of data from 338 patients and 10 included studies. In this review, a MESS (Mangled Extremity Severity Score) > 7 predicted primary amputation with a sensitivity of 0.95 (95% CI 0.69–0.99) and a specificity of 0.81 (0.65–0.91) [31]. Because of the limited specificity, the predictive accuracy of a MESS score > 7 is likely insufficient as the sole decision-making criterion for amputation of severely injured upper extremities. Conversely, a MESS score < 7 may predict the successful preservation of severely injured upper extremities. Currently, other scoring systems lack sufficient evidence to be considered in amputation decision-making [31].

In cases of subtotal amputation injuries, fracture stabilization and reconstruction of nerves, vessels, and soft tissues could be performed immediately after the resuscitation phase and stabilization of life-threatening injuries, with limb shortening if necessary. In total amputation injuries, the feasibility and condition of the amputated limb determine whether replantation or definitive amputation to create a viable stump is performed. Highly contaminated or severely open fractures, per se, do not constitute an indication for primary amputation in polytraumatized patients [33]. In these cases, stabilization and debridement remain the primary focus [33, 34].

These recommendations highlight the need for multidisciplinary decision-making and shared decision-making, as well as further research into optimal timing, predictive scoring, and long-term functional outcomes to guide evidence-based care in complex upper extremity trauma. Ultimately, however, the decision regarding the management of an extremity injury remains dependent on the patient’s current physiological condition and the clinical assessment of the situation by the treating multidisciplinary team.

3. In cases of arterial injury to the upper arm, revascularization should be performed as early as possible.

The recommendation for the management of vascular injuries is based on data from a U.S. registry study including 5,407 patients. This study demonstrated a significantly higher amputation rate when operative revascularization was performed after more than 90 min compared to less than 90 min (*p* = 0.007 in a time-to-event analysis) [28].

When interpreting this study, it should be noted that no risk adjustment was performed and the overall amputation rate was 1.8%. Restoration of adequate perfusion to the injured extremity remains a priority treatment step, even in polytraumatized patients. This is based on the evidence that ischemia duration is the critical, surgically modifiable factor influencing poor outcomes of the affected extremity [35–37].

Arterial injuries of the upper extremity represent a surgical emergency, and early revascularization is critical to optimize limb perfusion and prevent irreversible ischemic damage. Prompt restoration of blood flow reduces the risk of complications such as tissue necrosis, compartment syndrome, and secondary amputation. The timing and technique of revascularization should be carefully coordinated within the context of the patient’s overall condition, associated injuries, and hemodynamic stability. Multidisciplinary management involving general, vascular, and orthopaedic surgeons is essential to possibly achieve favourable functional outcomes while minimizing morbidity.

Recent studies even suggest reconsidering the traditional approach. In cases of fractures accompanied by vascular injury, particularly when critical ischemia times approach six hours, vascular reconstruction should be performed first, even before the application of the external fixator [38].

4. Upper extremity nerve injuries with discontinuity should be managed promptly, taking the patient’s overall condition into account. Management should adhere to the principles outlined in the S3 guideline “Treatment of Peripheral Nerve Injuries” (AWMF registry number 005/010).

Nerve injuries of the upper extremities should be addressed promptly, depending on the patient’s condition, and, if possible, in conjunction with definitive fracture stabilization. However, adequate evaluation of potential nerve lesions is often challenging, as polytraumatized patients are frequently intubated and ventilated upon hospital admission. Additionally, a thorough assessment of sensory and motor function in the fractured upper extremity is often not feasible at the accident site. For these reasons, the reported incidence of peripheral, fracture-associated nerve injuries of the upper extremity in the literature ranges from 1% to 18% [39, 40].

Unless the intervention is limited to decompression as part of fracture management, reconstruction of peripheral nerve injuries in the region of the long bones of the upper extremity is considered time-consuming and complex. Therefore, planned operative management in a stable environment should be prioritized [41–45]. Consequently, such a therapeutic approach should only be integrated into the primary management of polytraumatized patients in exceptional cases. This principle applies not only to individual peripheral nerve injuries but also to injuries of the upper brachial plexus [41–45].

Scientifically, evidence in this area is limited to small case series, which do not specifically focus on polytraumatized patients. Regarding outcomes, it is important to recognize that these are multifactorial and not solely determined by the timing of surgery [46].

For detailed guidance, reference should be made to the separate S3 guideline on the management strategies for peripheral nerve injuries [47, 48].

5. When nerve injuries with discontinuity are directly associated with a fracture, treatment should be performed in conjunction with definitive fracture stabilization.

Upper extremity nerve injuries with discontinuity should be managed promptly, with careful consideration of the patient’s overall condition and associated injuries. Management should follow the principles outlined in the S3 guideline “Treatment of Peripheral Nerve Injuries”, ensuring standardized, evidence-based care [47]. Early, guideline-adherent intervention can optimize functional recovery and reduce long-term disability, while balancing the patient’s physiological stability and treatment priorities.

Nerve injuries with discontinuity that occur in direct association with fractures present a particular therapeutic challenge. Coordinating nerve repair with definitive fracture stabilization allows for optimal alignment and immobilization, which may enhance the conditions for nerve regeneration and reduce the risk of secondary injury. This integrated approach also minimizes the need for multiple surgical interventions and may contribute to improved functional outcomes. However, this must always be considered in the context of the overall physiology of the severely injured patient.

The defined target population of this guideline comprises patients with multiple and/or severe injuries; however, the studies included in the systematic review demonstrate considerable heterogeneity with regard to injury patterns, trauma severity, patient characteristics, and clinical settings. Furthermore, only a proportion of the included cohorts adequately reflects a true polytrauma population as defined by established trauma criteria. This heterogeneity and indirectness must be carefully considered when interpreting the findings and assessing the transferability and generalizability of the recommendations to the broader population of critically injured polytrauma patients encountered in clinical practice.

### Limitations of the guideline

The recommendations of this S3 guideline are based on the best evidence available at the time of their development and on structured expert consensus where high-quality evidence was limited. The evidence base was limited by imprecision, with only few included studies and patients, and indirectness of the study populations. As is common in the field of polytrauma care, the available literature is characterized by a substantial heterogeneity of study designs, patient populations, injury patterns, and outcome measures, which restricts the generalizability of individual findings. Randomized controlled trials are scarce due to ethical, logistical, and methodological challenges inherent to trauma research, and many recommendations therefore rely on observational studies or expert opinion. Furthermore, rapid advances in trauma systems, diagnostic modalities, surgical techniques, and critical care management may limit the long-term applicability of certain recommendations. Variations in regional resources, institutional infrastructure, and interdisciplinary expertise may also influence the feasibility and implementation of specific guideline statements. Consequently, clinical judgment remains essential.

Regular updates of this guideline are necessary to incorporate emerging evidence and evolving standards of care in polytrauma management.

### Unanswered questions and future research

Despite advances in the management of upper extremity injuries in polytrauma patients, several important questions remain unresolved. The optimal timing and prioritization of definitive fixation relative to other life-threatening injuries are not well established. Evidence is limited regarding the best strategies for temporary stabilization, time and choice of fixation methods, and rehabilitation protocols in the context of multiple limb injuries.

Future research should include prospective, multicentre studies to evaluate functional outcomes, complication rates, and quality of life measures following different surgical and non-surgical approaches. Comparative studies on early versus delayed definitive fixation, and on various fixation techniques, are needed to guide evidence-based decision-making.

## Conclusion

The decision on the timing of surgical interventions for upper extremity injuries in polytrauma patients should be individualised, considering the patient’s overall physiological status, the nature of the injuries, and the potential risks and benefits of early versus delayed surgery. While early surgery can offer advantages in terms of functional recovery and reduced complications, it is imperative to ensure that the patient is adequately stabilised to tolerate the procedure. Decisions regarding limb salvage versus amputation should integrate the MESS score, with scores < 7 generally favouring preservation, whereas scores ≥ 7 require consideration of patient-specific factors such as comorbidities, functional demands, and preferences. Although the guideline addresses patients with multiple and/or severe injuries, the heterogeneity of the included studies and the limited representation of true polytrauma cohorts may restrict the generalizability of the findings to the broader critically injured trauma population.

## Data Availability

No datasets were generated or analysed during the current study.

## Abbreviations

AIS: Abbreviated injury scale
AMSTAR-2: Assessing the Methodological Quality of Systematic Reviews
AWMF: German Association of the Scientific Medical Societies
CI: Confidence interval
CRPS: Critical regional pain syndrome
CS: Constant-Murley Score
DASH: Disability of Shoulder, Arms, and Hand Questionnaire
GCS: Glasgow Coma Scale
GoR: Grade of recommendation
GPP: Good (clinical) practice point
GRADE: Grading of Recommendations Assessment, Development and Evaluation
HR: Hazard ratio
ICU: Intensive care unit
ISS: Injury Severity Score
MESS: Mangled Extremity Severity Score
PIC: Population, intervention, comparison, setting
PICO: Population, intervention, comparison, outcome
PICOS: Population, intervention, comparison, outcome, setting
PRISMA: Preferred Reporting Items for Systematic Reviews and Meta-Analyses
QUIPS: Quality In Prognosis Studies
RCT: Randomised controlled trial
REML: Restricted maximum likelihood
RIGHT: Reporting Items for practice Guidelines in Healthcare
ROBINS-I: Risk Of Bias In Non-randomized Studies-of Interventions
SF: Short form
SMFA: Short Musculoskeletal Function Assessment
VAS: Visual Analog Scale
